# Molecular and Genetic Landscape of Intravenous Leiomyomatosis: A Narrative Review

**DOI:** 10.1002/cai2.70075

**Published:** 2026-08-17

**Authors:** Lei Li, Jing Zhou, Jia Kang, Xudong Liu, Ran Chen, Meng Yuan, Zhuo Yu, Jinhui Wang

**Affiliations:** ^1^ 1National Clinical Research Center for Women's Health and Obstetric and Gynecologic Diseases, Department of Obstetrics and Gynecology Peking Union Medical College Hospital, Chinese Academy of Medical Sciences and Peking Union Medical College Beijing China; ^2^ Department of Geriatric Rehabilitation, Beijing Rehabilitation Hospital Capital Medical University Beijing China; ^3^ Laboratory Animal Research Facility, National Infrastructures for Translational Medicine, Institute of Clinical Medicine, Peking Union Medical College Hospital Chinese Academy of Medical Sciences and Peking Union Medical College Beijing China; ^4^ Department of Medical Oncology, Beijing Tsinghua Changgung Hospital, School of Clinical Medicine, Tsinghua Medicine Tsinghua University Beijing China

**Keywords:** benign smooth muscle tumor, genetic mechanisms, intravenous leiomyomatosis

## Abstract

Intravenous leiomyomatosis (IVL) is a rare benign smooth muscle tumor originating from the uterus and characterized by intravascular growth along the venous system, with potential extension to the inferior vena cava, right heart, or pulmonary arteries, leading to life‐threatening cardiorespiratory complications. IVL carries a 10%–31% recurrence rate and exhibits quasimalignant biological behavior despite its benign histology. Surgical resection is the main treatment. Current studies on IVL remain limited with regard to molecular and genetic mechanisms. This review systematically summarizes the research progress on IVL from the perspectives of chromosomal aberrations, copy number variations, gene mutations, transcriptomics, proteomics, histopathology, and epigenetic alterations. In addition, IVL is compared with uterine leiomyoma, leiomyosarcoma, and benign metastasizing leiomyoma to clarify its similarities and differences. This review also discusses unresolved issues, including tumor origin, intravascular invasive mechanisms and recurrence biomarkers, as well as prospects for future directions. A comprehensive understanding of the genetic and molecular features of IVL will help elucidate its pathogenesis, improve differential diagnosis and clinical management, and provide a theoretical basis for targeted therapy.

## Graphical abstract

Intravenous leiomyomatosis is a rare uterine benign tumor characterized by intravascular growth, quasi‑malignant behavior, and a high recurrence rate. This review summarizes its molecular and genetic research progress, compares it with similar tumors, discusses unsolved issues and prospects, contributing to its pathogenesis, diagnosis, and future treatment improvement.

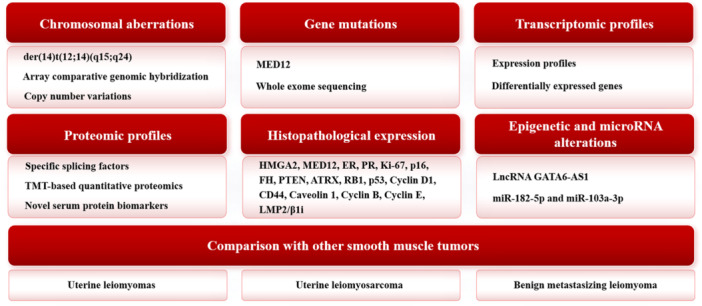

**Abbreviations**
aCGH: array comparative genomic hybridization
BML: benign metastasizing leiomyoma
CNV: copy number variation
DEGs: differentially expressed genes
DEPs: differentially expressed proteins
DPL: disseminated peritoneal leiomyomatosis.
ER: estrogen receptor
ECM: extracellular matrix
EU‐IVL: extra‐uterine IVL
FISH: fluorescence in situ hybridization
IVL: intravenous leiomyomatosis
LMS: leiomyosarcoma
LOH: loss of heterozygosity
MeSH: Medical Subject Headings
MSI: microsatellite instability
PR: progesterone receptor
SMC: smooth muscle cell
STUMP: smooth muscle tumors of uncertain malignant potential
stLM: soft tissue leiomyoma
U‐IVL: uterine IVL
UL: uterine leiomyomas
WES: whole exome sequencing
WGS: whole genome sequencing

## 1 Introduction

Intravenous leiomyomatosis (IVL) is a rare benign smooth muscle tumor that typically originates from the uterus, grows within or outside the pelvis, and extends along the venous system to the iliac vein, inferior vena cava, right heart, or even pulmonary arteries [1, 2, 3]. First described by Hirschfeld in 1896 [4], it predominantly affects premenopausal women, who typically have a history of uterine leiomyomas (ULs) [5]. The clinical presentation of IVL includes pelvic pain, abnormal bleeding, or life‐threatening cardiorespiratory symptoms due to vascular obstruction. When confined within the pelvis, the most common clinical manifestation is abdominal or pelvic pain accompanied by a pelvic mass and symptoms similar to those of UL. IVL also shares overlapping imaging features with uterine leiomyosarcoma (LMS), leading to frequent preoperative misdiagnosis [6]. If the tumor extends to the right heart or pulmonary arteries, patients may experience serious symptoms such as dyspnea, chest tightness, pulmonary embolism or even shock [7, 8]. The main treatment option for IVL is complete surgical resection. However, when the mass invades the venous system outside the uterus, the procedure is associated with an increased risk of intraoperative hemorrhage, collateral damage and mortality. The overall recurrence rate of IVL has been reported to be 10%–31% [5, 9]. Multiple recent studies have summarized the clinicopathological characterization of IVL. However, research on its molecular and genetic features is limited. In this review, we have summarized all the genetic and molecular research on IVL, aiming to elucidate its molecular mechanisms from the perspectives of chromosomes, the transcriptome, the proteome and epigenetics.

## 2 Literature Search Strategy

Literature retrieval was performed independently by two researchers in PubMed, Web of Science, and Embase from database inception until March 31, 2026. The search strategy utilized a combination of Medical Subject Headings (MeSH) terms and free‐text keywords related to IVL and its molecular and genetic characteristics. The core search string was ((“intravenous leiomyomatosis” OR IVL) AND (“genetics” OR “molecular mechanism” OR “chromosomal aberration” OR “gene” OR “copy number variation” OR “CNV” OR “transcriptome” OR “proteome” OR “protein”)). This strategy was adapted for the specific syntax of each database. Additionally, we manually screened the reference lists of all eligible articles to identify potentially missed publications.

All retrieved records were initially screened by title and abstract, followed by full‐text evaluation. The inclusion criteria comprised peer‐reviewed original studies, reviews and multiomics studies published in English that explored the molecular, genetic or pathological features of human IVL. We excluded duplicate articles, conference abstracts, case reports, letters, editorials, and studies focusing on clinical symptoms, imaging, or surgical treatment without exploration of mechanisms. Two authors independently assessed these studies, and discrepancies were resolved via group discussion to reduce selection bias. All final included articles are presented in the relevant parts of the following sections.

## 3 Molecular and on IVL

### 3.1 Chromosomal Aberrations and CNV of IVL

There is currently limited genetic research on IVL. In 2003, Dal Cin et al. [10] first reported two cases with a specific chromosomal abnormality, namely der (14)t (12;14)(q15;q24), in women diagnosed with IVL. The authors proposed that IVL originated from UL with the t(12;14)(q15;q24) translocation. An additional copy of 12q15‐qter and/or deletion of 14q24‐qter may be key genetic events leading to intravascular intrusion and proliferation. Fluorescence in situ hybridization (FISH) analysis also confirmed that the 12q breakpoint was located 5′ (centromeric) of *HMGA2*. In addition to chromosomal abnormalities, IVL also harbors recurrent regional chromosomal losses. The first array comparative genomic hybridization (aCGH) study of IVL was conducted in nine patients in 2014 by Buza et al. [11]. They revealed recurrent chromosomal losses, most frequently at 22q12.3–q13.1 (66.7%), followed by 22q11.23–q13.31 (55.6%), 1p36.13–p33 (33.3%), 2p25.3–p23.3 (33.3%), and 2q24.2–q32.2 (33.3%). These regions contained tumor suppressors such as *NF2*, *CHEK2*, *EWS*, *PDGFB*, *MAP3K7IP1*, *E2F2*, *ARID1A*, and *PTCH2*. Gains at 6p22.2, 2q37.3 and 10q22.2–q22.3 were also noted. Unlike UL, IVL exhibited complex and higher levels of genetic instability, with abnormal chromosomal segments overlapping with those of LMS, which may explain the clinically aggressive feature of IVL despite its benign histology (Table 1).

**Table 1:** Studies on chromosomal aberrations and copy number variations (CNVs) of IVL.

Authors	Year	Country	No. of patients	Methods	Main points
Dal Cin et al. [10]	2003	USA	2	Conventional cytogenetic karyotyping, FISH	IVL is characterized by chromosomal aberration der(14)t(12;14)(q15;q24). Extra copy of 12q15‐qter and/or loss of 14q24‐qter are critical genetic events.
Buza et al. [11]	2014	USA	9	aCGH	The most frequent copy number loss is 22q12.3–q13.1 (66.7%). IVL shows higher genetic instability overlapping with LMS.
Ordulu et al. [12]	2016	USA	12	Interphase FISH (*n* = 10), aCGH (*n* = 1)	HMGA2 expression is strongly linked to der(14)t(12;14)(q14.3;q24). IVL lacks 7q22 deletion.
Lu et al.[13]	2020	China	17 (12 UL, 17 uterine IVL, 12 extra‐uterine IVL	STR analysis (MSI/LOH)	IVL has a higher LOH rate, and uterine/extra‐uterine IVL likely arise independently rather than from single tumor spread.
Ordulu et al. [14]	2020	USA	26 (28 IVL lesions)	aCGH, hierarchical clustering, genomic index calculation	IVL divides into three molecular subgroups by recurrent deletions in 1p, 22q, and 10q. Genomic index does not predict aggressive behavior. Vascular morphology and 8q alterations may serve as potential prognostic markers for recurrence.
Yin et al. [15]	2025	China	8 IVL and normal myometrium	CNV analysis	Deletions are concentrated in 10q22.2, 10q24.32, 13q14, and 13q21‐31. Top gains include 8q24, 11q13, 12q24, 19q13 and 20q13. 10q deletion is closely related to the downregulation of proteins involved in focal adhesion and actin cytoskeleton regulation.

Abbreviations: aCGH, array comparative genomic hybridization; CNV, copy number variation; FISH, fluorescence in situ hybridization; IVL, intravenous leiomyomatosis; LMS, leiomyosarcoma; LOH, loss of heterozygosity; MSI, microsatellite instability; STR analysis, short tandem repeat analysis; UL, uterine leiomyomas.

Ordulu et al. [12] conducted interphase FISH on 10 IVL cases in 2016 and reported a strong correlation between *HMGA2* positivity and the co‐localization of *HMGA2* (12q14.3) and 14q24 hybridization signals (89.2% in positive cases vs. 12.4% in negative cases, *p* = 8.24 × 10^−10^), confirming a close association with the der(14)t(12;14)(q14.3;q24) chromosomal translocation. Four *HMGA2*‐positive cases showed *HMGA2* amplification. None of the cases showed the 7q22 deletion, which is commonly observed in UL [16]. Karyotyping of three cases identified characteristic t(12;14) translocations, including monosomy 22 in two cases. Consistent with previous research findings, aCGH detected complex CNVs with 14q24‐ > 14qter loss in one case. The authors concluded that *HMGA2* overexpression driven by t(12;14) was a key pathogenic event in IVL. Its quasimalignant intravascular invasive behavior may not only be mediated by t(12;14) alone but also by additional genetic alterations superimposed on *HMGA2* overexpression (such as monosomy 22 and complex CNVs). Lu et al. [13] investigated molecular alterations in 17 IVL cases, including 12 with concurrent UL, 17 with uterine IVL (U‐IVL), and 12 extra‐uterine IVL (EU‐IVL). Short tandem repeat analysis revealed microsatellite instability (MSI + ) in three samples (1 U‐IVL and 2 EU‐IVL) and loss of heterozygosity (LOH) in seven samples (1 UL, 3 U‐IVL, and 3 EU‐IVL). The LOH rate was significantly higher in IVL than in UL. Critically, the frequency of *MED12* mutations, MSI status, and LOH patterns differed between U‐IVL and EU‐IVL patients. U‐IVL and EU‐IVL may represent independent origins rather than clonal transmission from a single tumor, highlighting the clinical importance of radical surgical resection for patients with IVL.

Another aCGH was performed on 28 IVL tumors from 26 patients by Ordulu et al. [14] in 2020. The most frequent alterations involved 1p (39%), 22q (36%), 2q (29%), 1q (25%), 13q (21%), and 14q (21%). Unsupervised clustering revealed three distinct subgroups: Groups 1 and 2 (29% and 18% of cases, respectively) were defined by deletion of 22q, whereas Group 3 (18%) was characterized by 10q deletion. The median genomic index showed no significant difference between patients with IVL exhibiting aggressive behavior and those without such behavior. Notably, four of the five recurrent IVL cases had a vascular morphology, and three cases harbored 8q alterations. The authors then concluded that IVL might be driven by del(22q) or del(10q). Vascular morphology and 8q alterations may serve as potential prognostic markers for recurrence. Recently, Yin et al. [15] found that among eight IVL cases, the most frequent deletions were concentrated in 10q22.2, 10q24.32, 13q14 and 13q21‐31. The top gains included 8q24, 11q13, 12q24, 19q13 and 20q13. Notably, 10q deletion was closely related to the downregulation of proteins involved in focal adhesion and actin cytoskeleton regulation, which was a core genetic feature of IVL.

Based on the above research, the key genetic abnormalities of IVL have been identified. The der(14)t(12;14)(q15;q24) associated with *HMGA2* overexpression is a non‐random chromosomal translocation and critical pathogenic event. Recurrent chromosome loss (such as 22q, 1p, and 10q) and chromosome increase (such as 8q and 6p) are common and involve tumor suppressors. This complex genetic instability overlaps with LMS, which explains the invasive clinical behavior of IVL, despite its benign histology. IVL can be grouped on the basis of these genetic characteristics, and their recurrence markers can also be discussed. However, given the small sample size, these conclusions should be treated as references and provide direction for future research.

### 3.2 Gene Mutations and Dysregulation of IVL

IVL also has a unique gene mutation spectrum (Table 2). Buza et al. [11] used direct PCR sequencing in 2014 and reported no G > A transitions at *MED12* codon 44 (c.130/c.131) in any of the nine IVL cases, which is a common mutation in 80% of conventional UL cases. Wang et al. [17] investigated nine cases of IVL in 2020. Sanger sequencing of *MED12* exon 2 revealed two novel mutations in IVL: a synonymous mutation, c.141C > T (p.Asn47=), and an in‐frame deletion mutation, c.133_147del15 (p.Phe45_Pro49del). In contrast, one UL harbored a missense mutation, c.131G > A (p.Gly44Asp), at the hot‐spot codon 44 of *MED12* [19, 20]. IVL has *MED12* mutation patterns, distinct from those of UL. Similar results were seen in a study reported by Lu et al. [13] in 2020. Somatic *MED12* mutations (c.130G > A, p.G44S; c.131G > C, p.G44A) were significantly more frequent in UL (50%, 6/12) than in U‐IVL (0%, 0/17) and EU‐IVL (16.7%, 2/12), with no identical mutations observed between UL and IVL. *MED12* mutations may not act as a driver event in IVL. Two reviews by Zhou et al. [21] in 2023 and by Momeni‐Boroujeni et al. [1] in 2025 also reported that *MED12* mutations were rare in IVL (vs. 80% in UL), indicating distinct pathogenesis.

**Table 2:** Studies on gene mutations and dysregulation of IVL.

Authors	Year	Country	No. of patients	Methods	Main points
Buza et al. [11]	2014	USA	9	PCR sequencing for *MED12*	IVL lacks mutations at *MED12* codon 44 (c.130/c.131) common in UL.
Wang et al. [17]	2020	China	9	Sanger sequencing for *MED12* exon 2	*MED12* exon 2 hotspot mutations are rare in IVL; IVL and paired UL show distinct *MED12* mutation patterns, supporting IVL as a separate entity.
Lu et al. [13]	2020	China	17 (12 UL, 17 uterine IVL, and 12 extra‐uterine IVL	*MED12* sequencing	Somatic *MED12* mutations (c.130G > A, p.G44S; c.131G > C, p.G44A) are more frequent in UL (50%, 6/12) than in uterine IVL (0%, 0/17) and extra‐uterine IVL (16.7%, 2/12).
Li et al. [18]	2025	China	2 (1 BML, 1 IVL)	WES	BML and IVL share three mutated genes (*TCIRG1*, *KCNJ12*, *KCNJ18*) while having distinct overall mutation profiles. These shared mutations may contribute to their aggressive behavior.
Yin et al. [15]	2025	China	8 IVL and normal myometrium	WES	381 mutations in IVL. The most frequent types are nonsynonymous mutations (61.4%) and synonymous mutations (24.4%). 239 potential driver mutations are screened out after excluding synonymous mutations, with *PKD1*, *CAPN15*, *ZNF90*, *SNAPC4,* and *MUC4* as the prominently mutated genes.

Abbreviations: BML, benign metastasizing leiomyoma; IVL, intravenous leiomyomatosis; PCR, polymerase chain reaction; UL, uterine leiomyomas; WES, whole exome sequencing.

Whole‐exome sequencing (WES) of the intravascular and vaginal stump tumor tissues from one patient with IVL revealed 18 unique mutations (including those in *CADPS2*, *GPSM2*, *REEP4*, and *DUSP15*) [18]. Yin et al. [15] also performed WES in eight IVL and eight normal myometrium samples. They reported a total of 381 mutations in IVL, with nonsynonymous mutations being the most frequent type (61.4%), followed by synonymous mutations (24.4%). A total of 239 potential driver mutations were identified after excluding synonymous mutations, with *PKD1*, *CAPN15*, *ZNF90*, *SNAPC4* and *MUC4* as the prominently mutated genes (mutation frequency: 25.0%).

These studies indicate that IVL has a unique gene mutation spectrum that differs from that of UL, particularly in terms of *MED12* mutation. WES research is very rare, with only a few small‐sample studies exploring its mutation profile. Therefore, future studies should focus on large‐sample WES or whole‐genome sequencing (WGS) to validate the potential driver genes, elucidate the correlation between genetic mutations and clinical phenotypes, and further explore the molecular mechanisms underlying IVL pathogenesis and aggressive behavior.

### 3.3 Transcriptomic Profiles of IVL

Transcriptomic studies can also explain the overall characteristics of IVL (Table 3). Ordulu et al. [12] demonstrated from the expression profiles of three IVL cases that IVL was segregated from LMS rather than normal myometrium, conventional UL, or its histological variants. IVL and 9 UL with *t*(12;14) revealed 33 significantly altered genes (24 cancer‐related). Although IVL and UL had some similarities in histology and cytogenetics, IVL was more similar to LMS. This molecular similarity with malignant tumors provides support for classifying IVL's behavior as an “intermediate, quasimalignant” biological behavior.

**Table 3:** Studies on the transcriptomic profiles of IVL.

Authors	Year	Country	No. of patients	Methods	Main points
Ordulu et al. [12]	2016	USA	3	RNA expression profiling	IVL clusters transcriptionally with LMS, supporting its intermediate quasi‐malignant status.
Zhang et al. [22]	2019	China	5 IVL, 3 UL	RNA‑seq	IVL and UL share similar dysregulated pathways (hormone stimulus, ECM, cell adhesion); HOXA13 shows distinct expression and can serve as a biomarker to distinguish them.
Wang et al. [23]	2020	China	10 (5 IVL, 5 UL)	RNA‑seq	IVL displays distinct transcriptomic profiles from UL, with significant differences in angiogenesis and anti‑apoptosis genes, supporting IVL as a separate entity.
Zhang et al. [24]	2026	China	5 IVL and paired UL	Transcriptome sequencing	PIK3R1 is significantly upregulated in IVL and correlates with an immunosuppressive microenvironment; it can serve as a key immune biomarker and potential therapeutic target.

Abbreviations: ECM, extracellular matrix; IVL, intravenous leiomyomatosis; LMS, leiomyosarcoma; UL, uterine leiomyomas.

In 2019, Zhang et al. [22] performed RNA sequencing on five patients with IVL using multisite sampling (the distal, midpiece and proximal regions), three patients with UL, and their adjacent normal uterine muscle tissues. The authors identified 98 differentially expressed genes (DEGs) in IVL and 61 DEGs in UL compared with normal tissues, with both gene sets being functionally enriched in pathways related to steroid hormone stimulus, extracellular matrix (ECM), and cell adhesion. Samples from different IVL sites showed a low overlap rate of DEGs, suggesting evolutionary changes in gene expression during tumor growth. Unsupervised clustering analysis revealed a close transcriptional relationship between IVL and UL, which could not be completely separated, suggesting similar origins. Therefore, the authors summarized that IVL was closely related to UL and may be considered a subtype, although with distinct molecular features. Another RNA‐sequencing study reached a different conclusion, likely due to different comparison groups. Wang et al. [23] directly compared tissues from five patients with IVL and five patients with UL and identified 155 DEGs enriched in angiogenesis‐related and anti‐apoptosis‐related pathways in IVL. The gene expression profile of IVL was more complex and distinct from that of UL, supporting its classification as a lesion with uncertain malignant potential, partially due to increased angiogenesis and anti‐apoptotic mechanisms.

Recently, RNA sequencing from Zhang et al.'s [24] study on five cases of IVL and paired UL further focused on the up‐regulated gene *PIK3R1* and the down‐regulated gene *BMP4*. They revealed that *PIK3R1* was highly expressed in IVL, positively correlated with plasma cell abundance, and negatively correlated with follicular helper T cells and resting dendritic cells. The overall immune microenvironment of IVL was inhibited, making it difficult for tumor cells to be recognized and cleared by immune cells.

### 3.4 Proteomic Profiles of IVL

There are currently three studies on IVL proteomics (Table 4). Krasny et al. [25] performed the first proteomic analysis, aiming to compare this rare benign smooth muscle tumor (*n* = 3) to other smooth muscle tumors, including UL (*n* = 3), soft tissue leiomyoma (stLM, *n* = 7), and benign metastasizing leiomyoma (BML, *n* = 1). The key finding was that IVL was characterized by the coregulation of the expression of specific splicing factors, particularly those from the hnRNP, LSm, SR, and Sm classes. IVL was defined by a unique pattern of coregulated splicing factor expression, distinguishing it from other smooth muscle tumors. Yin et al. [15] further performed TMT‐based quantitative proteomics on eight pairs of IVL and normal myometrium samples, identifying 263 differentially expressed proteins (DEPs; 133 upregulated and 130 downregulated). These DEPs were mainly involved in focal adhesion, actin cytoskeleton regulation, and ECM‐receptor interaction pathways. Cytoskeletal and focal adhesion‐related proteins (vinculin, ACTIN1 and ACTA2) were significantly downregulated, whereas complement activation‐associated proteins were upregulated. Integrated multiomics analysis of proteomic and WES data identified 87 significantly correlated CNV‐DEP pairs, including 47 positive pairs enriched in ECM organization, cell‐matrix adhesion and integrin binding processes. Notably, 21.3% of these 47 CNV‐DEPs mapped to chromosome 10q, the deletion of which was associated with reduced expression of GLUD1, PDLIM1, SLK and vinculin. PDLIM1 and SLK participate in cytoskeletal and focal adhesion regulation [27, 28, 29]. Vinculin is the key focal adhesion‐related gene [30]. Thus, 10q deletion mediated the downregulation of proteins associated with the actin cytoskeleton and focal adhesion regulation, which may be a core molecular feature underlying IVL pathogenesis.

**Table 4:** Studies on the proteomic profiles of IVL.

Authors	Year	Country	No. of patients	Methods	Main points
Krasny et al. [25]	2022	UK	14 (3 IVL, 7 stLM, 3 uLM, 1 BML)	SWATH‑MS proteomics	IVL is distinguished by co‑regulated expression of spliceosome factors; two clusters (hnRNP/LSm/SR/Sm) are upregulated, with Cluster 3 linked to protein translocation and small GTPase signaling.
Yin et al. [15]	2025	China	8 IVL and normal myometrium	TMT proteomics	DEPs were mainly involved in focal adhesion, regulation of the actin cytoskeleton and ECM‐receptor interaction pathways. 10q deletion mediated downregulation of proteins associated with actin cytoskeleton and focal adhesion regulation.
Ge et al. [26]	2023	China	60 (30 IVL, 30 controls)	DIA proteomics	Four serum immunoglobulin‑related proteins serve as IVL progression biomarkers. One is a risk factor and three are protective, enabling non‑invasive prediction of recurrence.

Abbreviations: BML, benign metastasizing leiomyoma; DIA proteomics, data‐independent acquisition proteomics; ECM, extracellular matrix; IVL, intravenous leiomyomatosis; stLM, soft tissue leiomyoma; SWATH‑MS, sequential window acquisition of all theoretical fragments mass spectrometry; TMT proteomics, tandem mass tag (TMT)‐based quantitative proteomics; UL, uterine leiomyomas.

Ge et al. [26] identified novel serum protein biomarkers for IVL via DIA‐based proteomics in 60 cases (15 each for recurrent and non‐recurrent IVL, UL, and healthy controls). A total of 93 DEPs were found among 2582 proteins enriched in B‐cell‐mediated immune regulation. Integrated bioinformatics analyses revealed four hub proteins with good predictive values (AUCs of 0.68–0.69 and 0.92 for recurrent IVL), suggesting that these proteins are promising biomarkers for predicting the prognosis and progression of IVL.

Collectively, the results of these three proteomic studies have provided valuable insights into the molecular characteristics of IVL. Krasny et al. first revealed that IVL tumors were distinguished from other smooth muscle tumors by a unique co‐regulated expression pattern of splicing factors. Sheng et al. further identified chromosome 10q deletion as a core molecular feature, inducing downregulation of proteins related to the actin cytoskeleton and focal adhesion, thereby driving the pathogenesis of IVL. Ge et al. discovered promising serum protein biomarkers for IVL prognosis and progression prediction. Multiple cases highlight the importance of recognizing IVL's potential presentations and advocating for timely diagnosis and intervention [31]. These findings provide a solid foundation for further exploring the molecular mechanisms of IVL and optimizing its clinical diagnosis and management.

### 3.5 Histopathological Expression of Key IVL Genes

The expression of some key genes has also been histopathologically detected in IVL (Table 5). A series of 12 cases by Ordulu et al. [12] revealed that 58% (7/12) of IVL cases exhibited strong and diffuse nuclear expression of the HMGA2 protein, whereas no cases expressed MDM2 or CDK4. Immunohistochemical analysis by Lu et al. [13] in 2020 revealed no significant differences in the overexpression rate of HMGA2 or in the low expression rate of MED12 in UL, U‐IVL and EU‐IVL. The adjacent normal uterine smooth muscle cells invariably expressed low levels of HMGA2. Given that HMGA2 is overexpressed in both smooth muscle tumors but has low expression in normal myometrium, HMGA2 over‐regulation may act as an early event in the development of smooth muscle tumors.

**Table 5:** Histopathological expression of key IVL genes.

Authors	Year	Country	No. of patients	Methods	Main points
Ordulu et al. [12]	2016	USA	12	IHC	58% (7/12) of IVL exhibits strong and diffuse nuclear expression of HMGA2 protein. No cases express MDM2 or CDK4.
Lu et al. [13]	2020	China	17 (12 UL, 17 uterine IVL, and 12 extra‐uterine IVL	IHC for HMGA2 and MED12	No significant differences are found in the overexpression rate of HMGA2 or in the low‐expression rate of MED12 in UL, uterine IVL and extra‐uterine IVL. Adjacent normal uterine smooth muscle cells show HMGA2 low‐expression.
Wang et al. [17]	2020	China	9	IHC	All IVL and UL samples show positive staining for ER and PR, negative CD34, focal p16 expression, diffuse positivity for FH and PTEN, scattered ATRX expression, weak RB1 staining, and low positivity for p53 and Ki‐67 (both < 5%).
Ordulu et al. [14]	2020	USA	26 (28 IVL lesions)	IHC	IVL shows higher expression of p16 and Cyclin D1 compared to surrounding non‐neoplastic tissues. Cytoplasmic phosphorylated‐Rb is detected in all but one hyalinized IVL case. SMARCB1, FH, and SDHB expression is retained, and SOX10 and CAIX are negative.
Tamura et al. [32]	2021	Japan	2 IVL cases (plus 58 LMS, 52 UL, 74 normal)	IHC, CD44+ cell sorting, mouse xenograft metastasis model	CD44‐positive mesenchymal tumor stem‐like cells are detected in both IVL and LMS, while absent in UL. Expression of Cyclin E and Ki‐67 is rare in IVL (focal positivity < 5%), contrasting with high rates in LMS (> 50%). LMP2/β1i expression is defective in IVL and LMS, whereas strongly positive in normal myometrium.
Li et al. [18]	2025	China	2 (1 BML, 1 IVL)	IHC	The tumors express SMA, Desmin, h‐Caldesmon, ER, and PR, with a Ki‐67 proliferation index < 5%.
Zhang et al. [33]	2026	China	9	IHC	SMA, smooth muscle myosin heavy chain, Desmin, Caldesmon, ER and PR are strongly positive in IVL. The Ki‐67 index is mostly < 3%.

Abbreviations: BML, benign metastasizing leiomyoma; ER, estrogen receptor; IHC, Immunohistochemistry; IVL, intravenous leiomyomatosis; LMS, leiomyosarcoma; PR, progesterone receptor; UL, uterine leiomyomas.

Wang et al. [17] investigated nine cases of IVL in 2020. Histopathologically, IVL displayed an intravascular worm‐like growth pattern, lacked cytologic atypia and necrosis, and had low mitotic activity. In some cases, more prominent blood vessels and hyaline stroma were present than those observed in UL. All IVL and UL samples were positive for estrogen receptor (ER) and progesterone receptor (PR) but negative for CD34. Immunostaining revealed focal p16 expression, diffuse positivity for FH and PTEN, scattered ATRX expression, weak RB1 staining, and low positivity for p53 and Ki‐67 (both < 5%). Its immunophenotype is consistent with benign biological behavior. A similar conclusion was reached in the study by Li et al. [18] that one IVL case was composed of benign spindle smooth muscle cells without atypia, necrosis, or increased mitotic activity. The tumors expressed SMA, desmin, h‐caldesmon, ER, and PR, with a Ki‐67 proliferation index < 5%. Zhang et al. [33] also demonstrated that SMA, smooth muscle myosin heavy chain, desmin, caldesmon, ER and PR were strongly positive in IVL. The Ki‐67 index was mostly < 3%. The positive expression of ER and PR may partially explain why estrogen deprivation therapy, such as oophorectomy, can reduce the recurrence risk in patients with IVL [34].

Another study of 28 IVL cases showed significantly higher expression of p16 and cyclin D1 compared with surrounding non‐neoplastic tissues [14]. Cytoplasmic phosphorylated Rb was detected in all but one hyalinized IVL case, while SMARCB1, FH, and SDHB expression was retained, and SOX10 and CAIX expression was negative. These findings suggest that the Rb pathway (via p16/cyclin D1 overexpression and phosphorylated Rb) is involved in the pathogenesis of IVL. CD44, caveolin 1, cyclin B, cyclin E, Ki‐67, and LMP2/β1i staining was performed in 2 IVL, 58 LMS, and 52 UL tissue samples [32]. CD44‐positive mesenchymal tumor stem‐like cells were detected in both IVL and LMS but were absent in UL. The expression of cyclin E and Ki‐67 was rare in IVL (focal positivity < 5%), in contrast to high rates in LMS (> 50%). LMP2/β1i expression was defective in IVL and LMS but strongly positive in normal myometrium. These immunohistochemical findings indicate that IVL is histologically benign but exhibits overlapping malignant features with LMS.

### 3.6 Epigenetic and microRNA Alterations of IVL

Epigenetic studies of IVL were rare (Table 6). RNA‐sequencing analysis revealed that compared with UL, lncRNA *GATA6‐AS1* is a significantly downregulated epigenetic regulator in IVL [23]. This lncRNA is implicated in vascular disease and is potentially associated with the upregulation of the transcription factor GATA2 [36, 37].

**Table 6:** Epigenetic and microRNA alterations of IVL.

Authors	Year	Country	No. of patients	Methods	Main points
Wang et al. [23]	2020	China	10 (5 IVL, 5 UL)	RNA‑seq	LncRNA GATA6‐AS1 is a significantly downregulated epigenetic regulator in IVL compared with UL.
Barnaś et al. [35]	2023	Poland	12	RT‑qPCR	miR‑182‑5p is significantly upregulated in IVL compared to intramural leiomyomas. Both miR‑182‑5p and miR‑103a‑3p are elevated in IVL lesions from a single longitudinal patient, supporting their role in IVL pathogenesis.

Abbreviations: IVL, intravenous leiomyomatosis; RT‑qPCR, reverse transcription quantitative polymerase chain reaction; UL, uterine leiomyomas.

Barnaś et al. [35] investigated the role of two epigenetic factors, the oncomiRs miR‐182‐5p and miR‐103a‐3p. Key results showed that, compared with the intramural leiomyomas (*n* = 9), miR‐182‐5p expression was significantly elevated in both U‐IVL (*n* = 6) and EU‐IVL (*n* = 5). Both miR‐182‐5p and miR‐103a‐3p expression levels were significantly higher in IVL samples than in intramural samples collected from one woman during a 4‐year observation period. miR‐182‐5p was associated with the processes of intravasation and intravenous proliferation in IVL, whereas miR‐103a‐3p may be age‐ or hormone‐status dependent. This is the first report to link these specific oncomiRs to the intravenous spread of benign leiomyoma.

### 3.7 Integrative Multiomics and Molecular Regulatory Network

**Figure 1:**
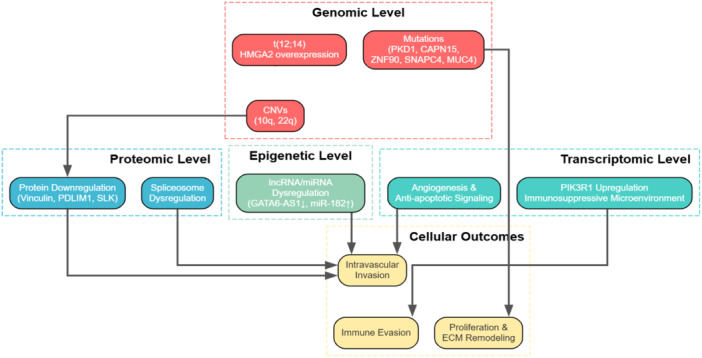
Integrative multiomics regulatory network of IVL. The pathogenic cascade of IVL is initiated by der(14)t(12;14)(q14.3;q24) translocation and subsequent HMGA2 overexpression. Recurrent copy number losses (especially 10q and 22q deletions) downregulate focal adhesion and actin cytoskeleton‐related proteins, enabling cell detachment and intravascular invasion. Distinct driver mutations (*PKD1*, *CAPN15*, *ZNF90*, *SNAPC4*, and *MUC4*), transcriptomic reprogramming (angiogenesis, anti‐apoptosis, and *PIK3R1*‐mediated immunosuppression), proteomic disorders (spliceosome dysregulation and cytoskeletal impairment), and epigenetic modifications (lncRNA GATA6‐AS1 and miR‐182) act together to drive intravascular growth, progression, and recurrence. This integrated network underlies the quasimalignant biological behavior of IVL. A schematic diagram was generated via Graphviz (https://graphviz.org), generated using GraphvizOnline online editor (https://dreampuf.github.io/GraphvizOnline/).

Integrated analysis across chromosomal aberrations, transcriptomes, proteomes, and signaling pathways reveals a stepwise molecular cascade that drives the initiation, intravascular invasion, progression, and recurrence of IVL. At the genomic level, the most upstream event appears to be the non‐random chromosomal translocation t(12;14)(q15;q24), which drives *HMGA2* overexpression. Recurrent CNVs, most notably at 10q and 22q, act as critical secondary hits, downregulating proteins (such as vinculin, PDLIM1, and SLK) involved in focal adhesion and actin cytoskeleton regulation, thereby weakening cell‐matrix attachment and enabling cellular migration and vascular invasion. Unlike UL, IVL lacks common *MED12* mutations and instead harbors potential mutations in *PKD1*, *CAPN15*, *ZNF90*, *SNAPC4*, and *MUC4*, which promote proliferation and ECM remodeling. Transcriptomic dysregulation increases angiogenesis and antiapoptotic signaling, whereas upregulated *PIK3R1* induces an immunosuppressive microenvironment to facilitate immune evasion. Proteomic abnormalities include aberrant expression of spliceosome factors and impaired cytoskeletal function. Epigenetically, downregulation of the lncRNA *GATA6‑AS1* and upregulation of the expression of miR‑182‑5p/miR‑103a‑3p further promotes intravascular growth (Figure 1). This hierarchical network defines IVL as a quasimalignant intermediate neoplasm and supports biomarker identification and the development of targeted therapies.

## 4 Comparison With Other Smooth Muscle Tumors

### 4.1 Compared With UL

IVL presents clinical symptoms similar to those of UL. Unlike common leiomyomas, which rarely invade blood vessels, IVL is characterized by intravascular growth with potential pelvic or extrapelvic extension and harbors CD44‐positive mesenchymal tumor stem‐like cells and a lack of LMP2/β1i cells, indicating that it has oncological properties similar to those of malignant LMS [9, 32].

In terms of chromosomal abnormalities, IVL shared the t(12;14)(q15;q24) translocation and a centromeric 12q breakpoint to *HMGA2* with UL, indicating its pathogenetic origin from leiomyomas carrying this translocation [10]. Ordulu et al. [12] additionally demonstrated that IVL was cytogenetically related to UL, as both harbor the t(12;14)(q15;q24) translocation, which drives *HMGA2* overexpression even more frequently in IVL (58%) than in UL (32%).

There are also significant differences between IVL and UL regarding the *MED12* mutation. Unlike typical ULs, which harbor frequent *MED12* codon 44 mutations, all nine IVL cases reported in Buza's study in 2014 showed a wild‐type *MED12*, indicating a distinct genetic pathogenesis from that of *MED12*‐mutant leiomyomas [11]. IVL may be biologically associated with *MED12*‐wild‐type leiomyomas carrying chromosomal alterations. Ordulu et al. [12] further confirmed that IVL lacks *MED12* mutations and 7q22 deletions. Wang et al. [17] revealed that IVL and UL also differed in *MED12* exon 2 mutation patterns: UL often harbored hot‐spot missense mutations (such as c.131G > A and p.Gly44Asp), whereas IVL rarely had *MED12* mutations. Lu et al. also reported that IVL and UL shared morphological similarity and high HMGA2 overexpression rates (75%–82.4%) but differed significantly in *MED12* mutation frequency [13]. More than 50% of ULs harbored *MED12* mutations [38], while U‐IVL showed no mutations, and EU‐IVL had a low mutation rate (16.7%).

In addition, patients with IVL exhibited higher genetic instability, a higher LOH frequency and occasional MSI, and overlapping molecular features with UL, such as recurrent del(22q) and 14q alterations, supporting its distinct molecular pathogenesis and quasimalignant intravascular growth behavior [14, 16].

By RNA sequencing, Zhang et al. [22] proposed that IVL is closely related to UL at the transcriptional level. Both exhibited hormone‐related pathogenic features, shared functional enrichment of DEGs in ECM and cell adhesion pathways, and clustered together, supporting the hypothesis that IVL might be a subtype of UL. RNA‐sequencing analysis by Wang et al. [23] revealed that, compared with UL, IVL had a far more complex gene expression profile, with significant dysregulation in angiogenesis and anti‐apoptosis pathways, whereas UL exhibited fewer DEGs and no characteristic alterations in these tumorigenic pathways.

IVL and UL share similar immunophenotypes—both are positive for ER and PR, negative for CD34, and have low Ki‐67 indices (< 5%) and focal p16 expression [17]. IVL also shows opposite HOXA13 expression patterns to UL, reflecting its unique quasimalignant biological behavior [22, 39].

Single‐cell sequencing analysis of a pair of IVL and UL revealed that IVL was dominated by fibroblasts (84.73%), with extremely low proportions of smooth muscle cells (SMCs, 4.38%) and endothelial cells (2.37%) [15]. In contrast, ULs were mainly composed of endothelial cells (56.64%) and SMCs (37.02%), while fibroblasts accounted for only 5.35%. These findings suggested that IVL exhibited hypovascular characteristics and was significantly different from UL in terms of cellular origin. The proportions of hydropic change and hyalinization in IVL were significantly higher than those in normal myometrium and UL (*p* < 0.001). The median vascular density was significantly lower than that of normal myometrium. These processes are closely correlated with the massive proliferation of fibroblasts and excessive deposition of collagen. The predominant fibroblasts in IVL may be the core pathological cells driving tumor progression. These fibroblasts highly express ECM‐related genes such as fibronectin, while the expression of SMC markers (ACTA2 and ACTIN1) is markedly suppressed. To date, two main hypotheses regarding the cellular origin of IVL remain controversial: one proposes that IVL derives from uterine SMCs that invade venous vessels, and the other hypothesis supports an origin from vascular wall SMCs. Current single‐cell and multiomics evidence challenges the traditional SMC‐origin theory and highlights the critical role of tumor‐associated fibroblasts in IVL pathogenesis. However, due to the limited sample size of single‐cell sequencing and the lack of cell lineage tracing experiments, the definitive origin cells of IVL have not been fully identified. Further single‐cell multiomics analysis and in situ verification are urgently required to clarify the cellular origin and intrinsic regulatory mechanisms of IVL initiation and progression.

In summary, IVL exhibits overlapping clinical and morphological features with UL but has distinct intravascular growth and quasimalignant biological behavior, indicating that IVL is a special subtype rather than a conventional smooth muscle tumor. At the molecular level, IVL is closely linked to UL through shared t(12;14)(q15;q24) translocation, *HMGA2* aberrations and hormone‑dependent pathways. However, there are significant differences in the absence of *MED12* mutations, high genetic instability, and unique transcriptomic and cellular profiles between IVL and fibroblasts. These molecular and cellular differences underlie its distinctive vascular invasiveness and may serve as potential diagnostic markers and therapeutic targets for distinguishing IVL from benign UL.

### 4.2 Compared With the LMS

IVL and LMS share notable oncological similarities, including the presence of CD44‐positive mesenchymal tumor stem‐like cells and LMP2/β1i‐deficient cells, indicating the potential for vascular invasion [32]. LMS is associated with high positive rates of cyclin E and Ki‐67 expression, poor prognosis, distant metastases to organs such as the lungs and liver, and a 5‐year survival rate of less than 20% [40]. IVL is a benign lesion with few cyclin E‐ and Ki‐67‐positive cells, no pathological signs of malignancy, and pelvic or extrapelvic extension of smooth muscle cells within blood vessels. Immunohistochemical staining also revealed that LMS frequently shows aberrant expression of ATRX, RB1, and PTEN and diffuse p53 positivity, whereas IVL retains intact expression of these tumor suppressor genes and shows only weak, scattered p53 staining, reflecting its benign biological behavior [17].

IVL and LMS both exhibit overlapping chromosomal aberrations involving chromosomes 1, 10, 12, 14 and 22, and both tumors display high genomic instability [10, 11, 41]. However, IVL lacks the additional genetic alterations required for the fully malignant phenotype seen in LMS, resulting in only quasimalignant behavior, such as invasive proliferation and metastatic potential. Ordulu et al. [14] reported that IVL exhibited molecular aberrations (such as del(10q) and 1p/13q deletions) that partially overlap with LMS and smooth muscle tumors of uncertain malignant potential (STUMP), but it has a far lower genomic index and lacks high‐grade cytology, brisk mitoses and genomic instability characteristic of LMS.

Gene expression analysis also revealed that, unlike ULs, which cluster separately in transcriptional profiling, IVLs segregate with LMSs according to hierarchical cluster analysis, and both IVLs and LMSs display complex genetic alterations; thus, IVLs are defined again as intermediate smooth muscle neoplasm [12].

### 4.3 Compared With BML

IVL harbors recurrent chromosomal alterations (1p, 22q, and 8q) that are also seen in rare benign leiomyoma including BML and disseminated peritoneal leiomyomatosis (DPL) [14, 42]. Its vascular subtype shares histological similarities with uterine angioleiomyoma but differs in its distinct genomic alteration profile.

IVL and BML share histological similarity with UL, as they display benign spindle cells that are positive for SMA/desmin/ER/PR and have a low Ki‐67 index [18, 43]. However, they exhibit distinct aggressive behaviors: intravascular extension for IVL and distant metastasis for BML. IVL and BML also share missense mutations in *TCIRG1*, *KCNJ12*, and *KCNJ18* [18]. These shared genes are implicated in tumor invasion, tumor migration, and ECM degradation [44, 45]. These findings suggest that IVL is a unique uterine‐derived smooth muscle tumor with benign histology but quasimalignant intravascular growth and that its aggressive behavior may be driven by specific genetic alterations shared with other similar tumors, such as BML.

## 5 Current Limitations and Potential Clinical Applications

Many studies focus on case reports and retrospective studies [46, 47, 48, 49]. According to a retrospective summary of 672 cases of IVL, 61% were confined to the pelvic cavity, and 35% extended beyond the renal veins (including intracardiac extension) [50]. Despite these preliminary findings, research on the genetic and molecular mechanisms of IVL remains relatively limited. Due to the insufficient sample size and inadequate research depth in existing studies, the majority of molecular conclusions are drawn from limited clinical specimens, which restricts the generalizability of these findings. The exact cellular origin of IVL and the detailed molecular mechanisms driving intravascular invasion have not been fully elucidated. There is no evidence on whether IVL originates from the uterine myometrium or vascular smooth muscle. Current multiomics studies are still in the preliminary stage, and single‐cell multiomics data and epigenetic regulatory networks require further in‐depth exploration, which hinders the in‐depth understanding of IVL pathogenesis.

Second, cellular and animal models dedicated to exploring the mechanisms of IVL are still lacking. The molecular and genetic alterations summarized above are mainly verified by conventional methods, including immunohistochemistry, Sanger sequencing and RT‐PCR. To date, only one study has conducted relevant cellular experiments and successfully isolated a CD44‐positive cell population from the human LMS cell line SK‐LMS‐1, which was identified as mesenchymal tumor stem‐like cells. In vivo experiments using nude mouse xenograft models demonstrated that compared with CD44‐negative cells, CD44‐positive stem‐like cells did not strongly affect primary tumor growth. However, these cells possessed a markedly stronger capacity to form hematogenous micrometastases in alveolar tissues. These findings confirmed that CD44‐positive mesenchymal tumor stem‐like cells have strong potential for vascular invasion and hematogenous metastasis. Notably, similar CD44‐positive mesenchymal tumor stem‐like cells were also detected in IVL, which explains the phenotypic similarity between the intravascular growth of benign IVL and the metastatic tendency of malignant LMS. Unfortunately, there have been no reports on stable IVL cell lines, specialized animal models, or functional validation studies focusing on core molecular alterations. This gap represents a major limitation in current IVL research. Future studies are urgently needed to establish standardized in vitro cell models, patient‐derived xenograft models and organoid models and to conduct systematic functional experiments to clarify the regulatory roles of these genetic changes in IVL pathogenesis, intravascular invasion and recurrence.

Third, the identification of reliable recurrence biomarkers for IVL is still in its early stages. The current findings are primarily based on retrospective studies with very small sample sizes, and their predictive value has not been validated by large‐scale prospective studies. Postoperative management of IVL is complex, particularly in patients with vascular involvement. Multidisciplinary collaboration, including preoperative planning, postoperative surveillance, and recurrence prediction and patient stratification, is crucial in IVL management, ensuring a comprehensive approach that optimizes patient outcomes [51]. Therefore, it is necessary to conduct well‐designed prospective cohort studies to confirm the correlation between these molecular indicators and IVL recurrence and to identify additional biomarkers that can accurately predict disease recurrence and guide clinical follow‐up and treatment strategies.

Despite these limitations, the accumulated molecular and genetic findings of IVL have yielded promising translational value for clinical practice. The distinct molecular profiles that effectively distinguish IVL from UL and LMS may include the presence of der(14)t(12;14)(q14.3;q24) translocation and *HMGA2* overexpression, combined with the absence of typical *MED12* hotspot mutations, which may serve as reliable molecular biomarkers. Immunohistochemical phenotypes and CD44‐positive mesenchymal tumor stem‐like cells also help distinguish IVL from malignant LMS. For prognostic evaluation and recurrence monitoring, chromosomal alterations, including 10q deletion, 8q aberrations, and vascular morphological characteristics are correlated with aggressive phenotypes and postoperative recurrence of IVL. Additionally, serum protein biomarkers identified via proteomic analysis provide a noninvasive approach for long‐term follow‐up of patients. With respect to therapeutic strategies, the positive expression of ER and PR supports the application of estrogen deprivation therapy to reduce recurrence risk, whereas dysregulated *PIK3R1* and abnormal signaling pathways related to focal adhesion and cytoskeleton regulation offer potential targets for future targeted therapy. Future research may also involve integrated multiomics analyses, combining genomics, proteomics, and epigenetics, which hold great promise for exploring key targets and molecular mechanisms underlying the vascular invasiveness of IVL and for identifying novel and specific diagnostic biomarkers for IVL. In addition, the establishment of specific preclinical models, including patient‐derived xenografts and organoid models, which are essential for validating molecular mechanisms, screening targeted therapeutic agents, and evaluating treatment responses, is urgently needed.

## 6 Conclusion

IVL is a molecularly distinct tumor characterized by benign histological features and malignant intravascular invasive behavior. It shares certain genetic similarities with benign UL and malignant LMS, and exhibits its intermediate, quasimalignant biological characteristics. However, current research on IVL remains limited, with a lack of systematic exploration of its core pathological and molecular mechanisms, largely because of the limited sample size. Future investigations should focus on more comprehensive and in‐depth research on the origin and intravascular invasive mechanisms of IVL, as well as prevention and treatment strategies. Clarifying these key aspects will not only deepen our understanding of IVL pathogenesis but also provide evidence for developing standardized clinical management strategies and improving patient outcomes.

## Author Contributions

**Lei Li:** writing – original draft (lead), writing – review and editing (equal), investigation (equal), funding acquisition (equal). **Jing Zhou:** investigation (lead), writing – original draft (equal), writing – review and editing (equal). **Jia Kang:** investigation (supporting), writing – review and editing (lead). **Xudong Liu:** investigation (supporting), writing – review ans editing (equal). **Ran Chen:** data curation (equal), investigation (equal), writing – review and editing (supporting). **Meng Yuan:** data curation (equal), investigation (equal), writing – review and editing (supporting). **Zhuo Yu:** supervision (lead), project administration (lead), writing – review and editing (equal), funding acquisition (equal). **Jinhui Wang:** supervision (lead), writing – review and editing (equal), project administration (equal).

## Ethics Statement

The authors have nothing to report.

## Consent

The authors have nothing to report.

## Conflicts of Interest

Professor Zhuo Yu is a member of the *Cancer Innovation* Editorial Board. To minimize bias, he was excluded from all editorial decision‐making related to the acceptance of this article for publication. The remaining authors declare no conflicts of interest.

## Data Availability

The data that support the findings of this study are available from the corresponding author upon reasonable request.
